# Trust in Artificial Intelligence-Generated Oral Health Information and Its Influence on Patient Decision-Making: A Cross-Sectional Study

**DOI:** 10.3290/j.ohpd.c_2805

**Published:** 2026-09-02

**Authors:** Berrak Ulu Emir, Bilkan Kara, Sayna Behkar, Erkan Özcan

**Affiliations:** a Berrak Ulu Emir Researcher, Department of Periodontology, Gulhane Faculty of Dentistry, University of Health Sciences, Ankara, Turkey. Drafted the manuscript, collected the data, and approved the final version of the manuscript.; b Bilkan Kara Researcher, Department of Periodontology, Gulhane Faculty of Dentistry, University of Health Sciences, Ankara, Turkey. Contributed to the study design, critically revised the manuscript, and approved the final version of the manuscript.; c; d Sayna Behkar PhD Student, Department of Periodontology, Gulhane Faculty of Dentistry, University of Health Sciences, Ankara, Turkey. Collected the data, approved the final version of the manuscript.; e Erkan Özcan Researcher, Department of Periodontology, Gulhane Faculty of Dentistry, University of Health Sciences, Ankara, Turkey. Critically revised the manuscript and approved the final version of the manuscript.

**Keywords:** artificial intelligence, ChatGPT, oral health, survey, trust

## Abstract

**Purpose:**

Artificial intelligence (AI)-assisted applications are increasingly used to obtain health-related information; however, concerns remain regarding the reliability of AI-generated responses and their influence on patient attitudes and decision-making. This study aimed to evaluate the use of AI-assisted applications among patients, their trust in AI-generated information, and the influence of these applications on oral-health-related decision-making.

**Methods and Materials:**

This cross-sectional questionnaire-based study included 430 patients attending the Department of Periodontology. Participants completed a structured questionnaire evaluating AI-assisted application use, trust in AI-generated oral health information, and the effects of AI on oral-health-related attitudes. Categorical variables were presented as frequencies (n) and percentages (%). Associations between categorical variables were analysed using the chi-square test. Effect sizes were evaluated using Cramér’s V coefficient. Statistical significance was set at *p* < 0.05.

**Results:**

A total of 430 participants completed the questionnaire, including 300 AI users. AI usage rates were generally similar across reasons for admission, although the highest usage was observed among individuals attending routine check-ups (18/19; 94.7%). In treatment-related decisions, most participants preferred dentists’ opinions over AI-generated recommendations (80.8%; *p* < 0.01). Trust was significantly associated with AI usage frequency (*p* < 0.01), whereas education level was significantly associated with AI usage (*p* < 0.001). Education level was also associated with willingness to consult AI-assisted programmes regarding dentist-created treatment plans (*p* = 0.033).

**Conclusion:**

AI-assisted applications have become an important part of health information-seeking behaviour, particularly among younger and more educated individuals. However, patients remain cautious about the reliability of AI-generated information and continue to prioritise professional dental expertise in treatment-related decisions.

The rapid growth of artificial intelligence (AI) is changing how people access health-related information, creating new alternatives to traditional patient–clinician communication.16 Although AI-assisted tools such as ChatGPT (Chat Generative Pre-trained Transformer, OpenAI, San Francisco, CA, USA) and Gemini (Google, Mountain View, CA, USA) facilitate easier access to medical and dental information, concerns remain regarding the accuracy, reliability, and consistency of the information generated by these systems.^9,27
^ Despite these concerns, individuals may frequently use these tools while still being sceptical about the reliability of the information they provide.^1^ This issue is particularly important in clinical settings, where inaccurate or misunderstood information may affect health-related choices and patient attitudes toward treatment.^10^


Research suggests that the usage of AI technology is associated with demographic characteristics.^5^ Factors such as age, education level, and digital literacy skills influence the frequency and duration of technology use.^11^ Generally, younger individuals and those with higher education or stronger digital skills tend to use AI more frequently.^8,32
^ However, frequent use does not necessarily mean greater trust, as many users still question the reliability of AI-generated health information.^17^


In dentistry, patient decision-making is often shaped by both subjective symptoms and professional clinical assessment.^13^ Therefore, it is particularly important to understand how AI influences patients’ information-seeking behaviour in this context.^34^ Although the contribution of AI as a supporting tool cannot be overestimated, its impact on such decisions as visiting the dentist, confidence in dentists, and accepting recommended treatments still needs to be clarified.^20^ Existing literature supports the view that health professionals, rather than AI, are still the leading source of reliable information.^23,26
^ However, evidence regarding how patients perceive and use AI-generated oral health information in making oral-health-related decisions remains limited.

The aim of this study was to evaluate the frequency of AI-assisted application use among patients attending the Department of Periodontology, their perceived trust in information obtained from these applications, and the influence of AI-generated responses on oral-health-related decision-making processes. Additionally, the study aimed to investigate the effects of positive and negative AI-generated information on patients’ anxiety and reassurance levels. The possible associations between demographic characteristics, AI usage habits, and trust perception were also assessed as secondary objectives of the study.

## Methods and Materials

### Study Design and Participants

The study was designed as a cross-sectional, questionnaire-based study and received ethical approval from the University of Health Sciences Gulhane Training and Research Hospital Clinical Research Ethics Committee (approval no: 2026/109). The study was conducted in accordance with the principles of the Declaration of Helsinki (as revised in 2013). The study population consisted of patients attending the Department of Periodontology, Gulhane Faculty of Dentistry, University of Health Sciences, Ankara, Turkey, in April 2026. A total of 430 participants completed the questionnaire. Of these, 300 participants reported using AI-assisted applications and constituted the AI-user subgroup. An *a priori* power analysis was performed for this subgroup because the primary analyses focused on participants who used AI-assisted applications. All participants were informed about the study and provided written informed consent prior to participation. Data collection was performed anonymously, and no personally identifiable data were recorded. A consecutive sampling strategy was used, and all eligible patients attending the Department of Periodontology during the study period were consecutively invited to participate until the required sample size for the AI-user subgroup was reached.

Data were collected using a structured self-administered questionnaire completed by the participants during their clinical visits. The questionnaires were distributed by the clinic secretary to eligible participants, who completed them independently before their clinical examination. The researchers were not present during questionnaire completion and therefore did not influence participants’ responses, minimising the potential for social desirability bias. Completed questionnaires were subsequently reviewed by a single researcher for completeness and consistency before data analysis. The questionnaire was administered to evaluate sociodemographic characteristics, oral health perceptions, frequency of AI-assisted application use, trust in AI-generated information, and the effects of this information on health-related decision-making processes. Participants eligible for inclusion were individuals aged 18 years or older, literate, and willing to participate voluntarily in the study. Participants who completed the questionnaire more than once, provided incomplete or inappropriate responses, or had mental disorders or cognitive dysfunctions were excluded from the study.

### Questionnaire Development

The questionnaire was developed specifically for this study and consisted of 22 items, including five items related to sociodemographic characteristics and three items related to clinical characteristics. The questionnaire was designed to evaluate participants’ use of digital information sources and AI-assisted applications, their perceptions regarding the reliability and appropriateness of AI-generated health information, and the influence of such information on oral-health-related attitudes and decision-making behaviours. To ensure a consistent understanding of the term, examples of AI-assisted applications, including ChatGPT, Google Gemini, Microsoft Copilot (Microsoft Corporation, Redmond, WA, USA), and Grok (xAI, Palo Alto, CA, USA), were provided within the questionnaire.

The questionnaire included items assessing the frequency of AI-assisted application use in daily life and for general and oral health purposes, trust in AI-generated information, attitudes toward the use of AI in health care, and preferences between AI-generated responses and professional dental opinions. In addition, behavioural and emotional responses associated with AI consultations were evaluated, including the influence of AI on decisions to seek dental care, treatment-related decisions, anxiety following negative AI-generated information, and reassurance following positive AI-generated information. The questionnaire also assessed participants’ opinions regarding the supportive role of AI in dental care and their tendency to verify dental treatment plans using AI-assisted applications.

Different response formats were used according to the nature of the questionnaire items. The item assessing the most frequently preferred digital information source was structured as a nominal categorical question, whereas items evaluating the frequency of AI-assisted application use were designed as ordinal frequency-based categories. Most of the remaining items assessing attitudes, perceptions, emotional responses, and decision-making behaviours were structured using five-point Likert-type response options.^24^ The complete questionnaire used in this study is provided in English as Supplementary Material (S1 File, available directly from the author).

As the questionnaire had a multidimensional structure and was not intended to measure a single latent construct, internal consistency analysis using Cronbach’s alpha was not considered appropriate. Before the main study, the questionnaire was pilot-tested in 20 patients attending the Department of Periodontology to assess the clarity, comprehensibility, and feasibility of the questionnaire items. Based on participants’ feedback, minor wording revisions were made to improve item clarity. Data obtained from the pilot study were not included in the final analysis. Content validity of the questionnaire was evaluated using the Content Validity Ratio (CVR) and Content Validity Index (CVI).

### Content Validity

The content validity of the questionnaire was evaluated by 10 experts using Lawshe’s technique. The CVR and CVI were calculated for each item.^21,29
^ For 10 experts, the minimum acceptable CVR value was 0.62. Items with scores below the required threshold were revised or excluded from the questionnaire. An Item-level Content Validity Index (I-CVI) value of 0.78 was also used as a criterion in excluding questions based on content validity. The questionnaire demonstrated good content validity, as indicated by a Scale-Level Content Validity Index/Average (S-CVI/Ave) of 0.856.

### Sample Size

An *a priori *power analysis was performed using (G*Power 3.1, Heinrich Heine University Düsseldorf, Germany), under the chi-square (χ^2^) testing paradigm to estimate the minimal number of subjects.^12^ The parameters used in this calculation include effect size (Cohen’s w) equal to 0.20, the alpha (α) equal to 0.05, the degrees of freedom (df) equal to 4, and the power (1−β) equal to 0.80. Based on these assumptions, the estimated minimum required sample size for AI users was calculated as 299 participants. Therefore, patient recruitment was continued until the target number of AI users was reached.

### Statistical Analysis

Statistical analysis was performed using a statistical software package (SPSS v.21.0, IBM Corp., Armonk, NY, USA). Descriptive statistics were calculated for categorical variables and presented as frequencies (n) and percentages (%). Distributions in cross-tabulations were evaluated using row percentages to improve interpretability.

Associations between categorical variables were analysed using the Pearson chi-square (χ^2^) test. In cases where expected cell frequencies were low, particularly when more than 20% of the cells had expected counts below 5, the Monte Carlo simulation method was used to improve the reliability of the analysis, and these results were indicated in the relevant tables.

To evaluate the magnitude of statistically significant associations, Cramér’s V effect size coefficient was calculated. Effect sizes were interpreted using conventional benchmarks, with values of 0.10–0.29 indicating a small effect, 0.30–0.49 indicating a moderate effect, and ≥ 0.50 indicating a large effect. It should be noted that these benchmarks do not account for differences in degrees of freedom across contingency tables. Statistical significance was set at *p* < 0.05 for all analyses.

## Results

A total of 430 participants completed the questionnaire, including 300 individuals who reported using AI-assisted applications. The sociodemographic characteristics of the patients are presented in Table 1. Age was found to be a primary determinant of AI adoption, with the highest usage in the 18–30 age group (88.3%) and a progressive decline in older cohorts. Gender showed a marginal difference, with females exhibiting slightly higher usage (71.8%) than males (67.0%). AI usage increased markedly with higher educational attainment. Usage rates increased from 36.8% among primary school graduates to 90.5% among participants with postgraduate degrees. Professionally, students reported the highest utilisation (90.2%), while retirees reported the lowest (45.7%) (Table 1).

**Table 1 Table1:** Sociodemographic characteristics of participants

Sociodemographic variables	AI user n (%)	Non-AI user n (%)	Total n (%)
Age groups			
Gender			
Education level			
Marital status			
Occupation			
18–30	151 (88.3%)	20 (11.7%)	171 (100%)
31–40	48 (64.0%)	27 (36.0%)	75 (100%)
41–50	38 (49.4%)	39 (50.6%)	77 (100%)
51 and above	63 (58.9%)	44 (41.1%)	107 (100%)
Female	176 (71.8%)	69 (28.2%)	245 (100%)
Male	124 (67.0%)	61 (33.0%)	185 (100%)
Primary school	14 (36.8%)	24 (63.2%)	38 (100%)
Middle school	15 (40.5%)	22 (59.5%)	37 (100%)
High school	96 (65.3%)	51 (34.7%)	147 (100%)
University	156 (83.4%)	31 (16.6%)	187 (100%)
Postgraduate	19 (90.5%)	2 (9.5%)	21 (100%)
Married	135 (59.0%)	94 (41.0%)	229 (100%)
Single	165 (82.1%)	36 (17.9%)	201 (100%)
Student	83 (90.2%)	9 (9.8%)	92 (100%)
Housewife	47 (52.8%)	42 (47.2%)	89 (100%)
Self-employed	13 (54.2%)	11 (45.8%)	24 (100%)
Public sector employee	61 (78.2%)	17 (21.8%)	78 (100%)
Private sector employee	51 (72.9%)	19 (27.1%)	70 (100%)
Retired	21 (45.7%)	25 (54.3%)	46 (100%)
Unemployed	24 (77.4%)	7 (22.6%)	31 (100%)
Note: Percentages represent row percentages (AI users vs. non-AI users within each category).Abbreviation: AI, artificial intelligence.			

No significant difference was found between previous treatment experience and AI use. Similarly, there was no difference between the presence of active dental treatment and AI use. AI usage rates were generally similar according to the reason for admission, although the highest usage was observed among individuals attending routine check-ups (18/19; 94.7%) (Table 2).

**Table 2 Table2:** AI usage according to clinical history and reason for clinic visit

Variable	Category	AI user n (%)	Non-AI user n (%)	Total n (%)
Previous treatment	Yes	97 (68.3%)	45 (31.7%)	142 (100%)
	No	203 (70.5%)	85 (29.5%)	288 (100%)
Active treatment	Yes	74 (69.8%)	32 (30.2%)	106 (100%)
	No	226 (69.8%)	98 (30.2%)	324 (100%)
Reason for clinic visit	Gum bleeding	25 (65.8%)	13 (34.2%)	38 (100%)
	Dental scaling	167 (69.3%)	74 (30.7%)	241 (100%)
	Halitosis	9 (64.3%)	5 (35.7%)	14 (100%)
	Gingival recession	25 (65.8%)	13 (34.2%)	38 (100%)
	Tooth mobility	12 (60.0%)	8 (40.0%)	20 (100%)
	Gum pain/swelling	26 (70.3%)	11 (29.7%)	37 (100%)
	Routine check-up	18 (94.7%)	1 (5.3%)	19 (100%)
	Referral by a dentist	18 (78.3%)	5 (21.7%)	23 (100%)
Note: Percentages are presented as row percentages (AI users vs non-AI users within each category).Abbreviation: AI, artificial intelligence.				

Among individuals using AI, the highest proportion was observed in the ‘very rarely’ usage group (37.7%). Nevertheless, the combined proportion of participants using AI several times a week and every day/almost every day was also notable (51.0%).

When the perceived reliability of information obtained from AI-assisted applications was evaluated, more than half of the participants responded ‘undecided’ (52.2%). Although the proportion of participants who considered AI-generated information reliable was higher than those who considered it unreliable (33.4% and 14.5%, respectively), uncertainty remained the predominant attitude (52.2%).

It was observed that the use of AI for obtaining general health-related information was mostly at a low frequency. The response ‘I use it very rarely’ accounted for more than half of the participants (50.5%). Nearly one-quarter of the participants reported never using AI for oral and dental health information (24.2%), while more than half stated that they used it very rarely (52.2%).

A noteworthy finding was that the largest proportion of participants stated that information obtained from AI did not influence their decision to consult a dentist (55.2%). The vast majority of participants (90.0%) reported that they would consult a dentist when experiencing an oral or dental health problem. While a substantial proportion of participants (44.4%) viewed AI-generated information as supportive of the data provided by dentists, more than half (52.8%) indicated they preferred a professional opinion over AI when making important health decisions.

This reliance on professional expertise was particularly evident in treatment planning; 46.8% of participants stated that they would not consider additionally consulting AI regarding a treatment plan proposed by their dentist, whereas 30% reported that they would consider consulting AI and 23.2% remained undecided. As shown in Figure 1, when faced with a situation where AI and a dentist provide different treatment plans, a significant majority (80.8%) of participants prioritised the dentist’s opinion. A statistically significant association was found between the general preference for dentists and prioritising dentists’ treatment plans over AI-generated recommendations (χ^2^ = 129.3; *p* < 0.01), with a moderate effect size (Cramér’s V = 0.33).

**Fig 1 Fig1:**
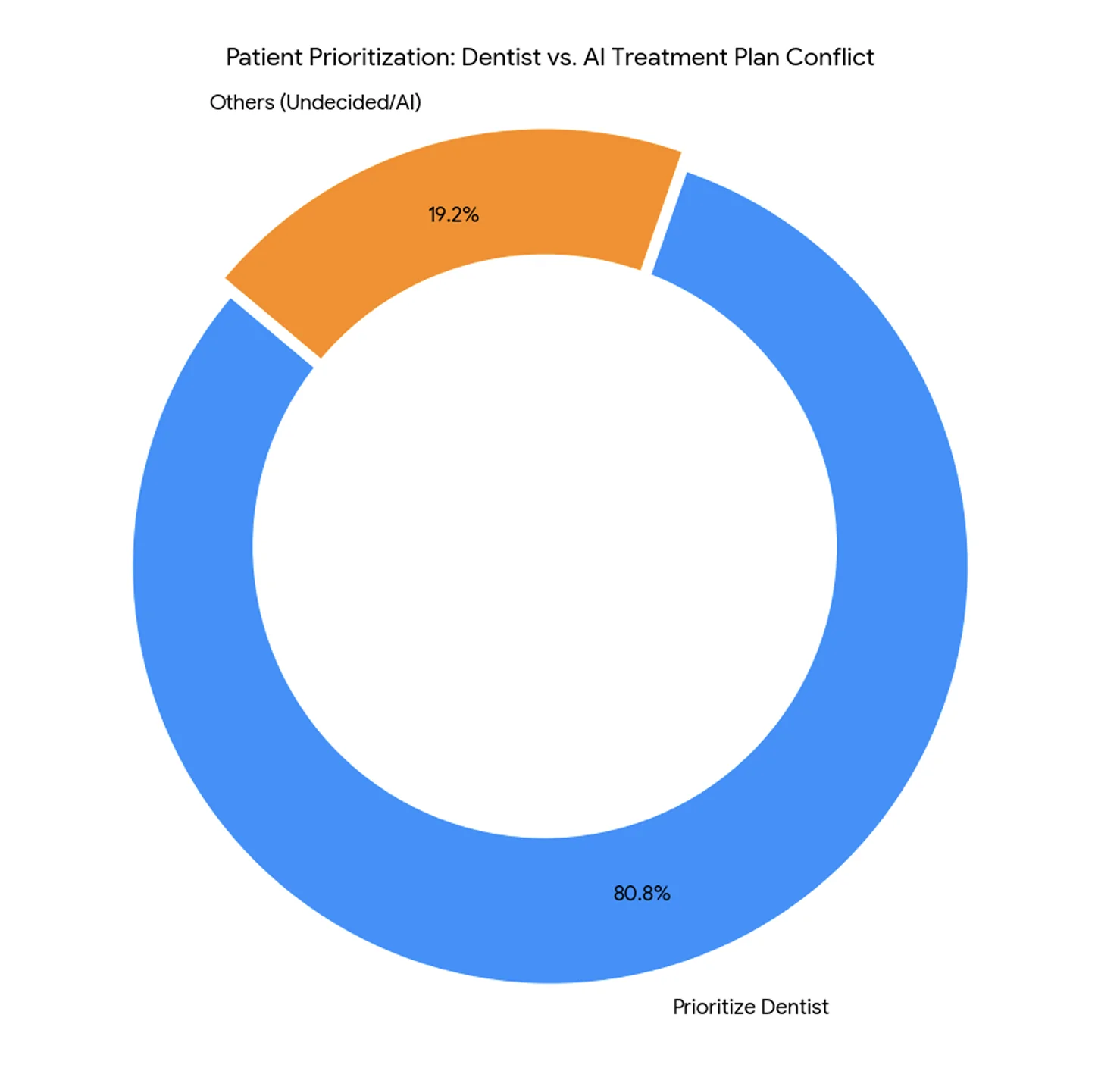
Participants’ prioritisation of dentists’ treatment plans and AI-generated recommendations when conflicting treatment plans were presented among AI users (n = 300). Percentages are shown (χ^2^ = 129.3, *p* < 0.01, Cramér’s V = 0.33).

The distribution regarding the effect of negative AI-generated information on anxiety demonstrated a balanced pattern, as the proportions of participants reporting increased anxiety and those reporting no increase in anxiety were similar (39.3% and 37.7%, respectively). Perceptions that positive AI-generated information had a reassuring effect were more common than perceptions indicating no reassuring effect (43.7% and 24.6%, respectively). However, the proportion of undecided individuals was also considerable in this context (31.6%).

An analysis of digital information-seeking behaviours revealed a statistically significant difference between AI users and non-users in the digital resources preferred for obtaining oral health information (χ^2^ = 82.7; *p* < 0.001; V = 0.44). As shown in Figure 2, search engines (e.g. Google, Yandex) were the most frequently preferred digital sources for obtaining oral-health-related information among AI users (40.7%), followed by AI-assisted applications (24.3%). In contrast, among non-AI users, the most frequently reported response regarding digital sources for obtaining oral health information was ‘not using any digital source’ (42.3%), compared to 9.7% among AI users.

**Fig 2 Fig2:**
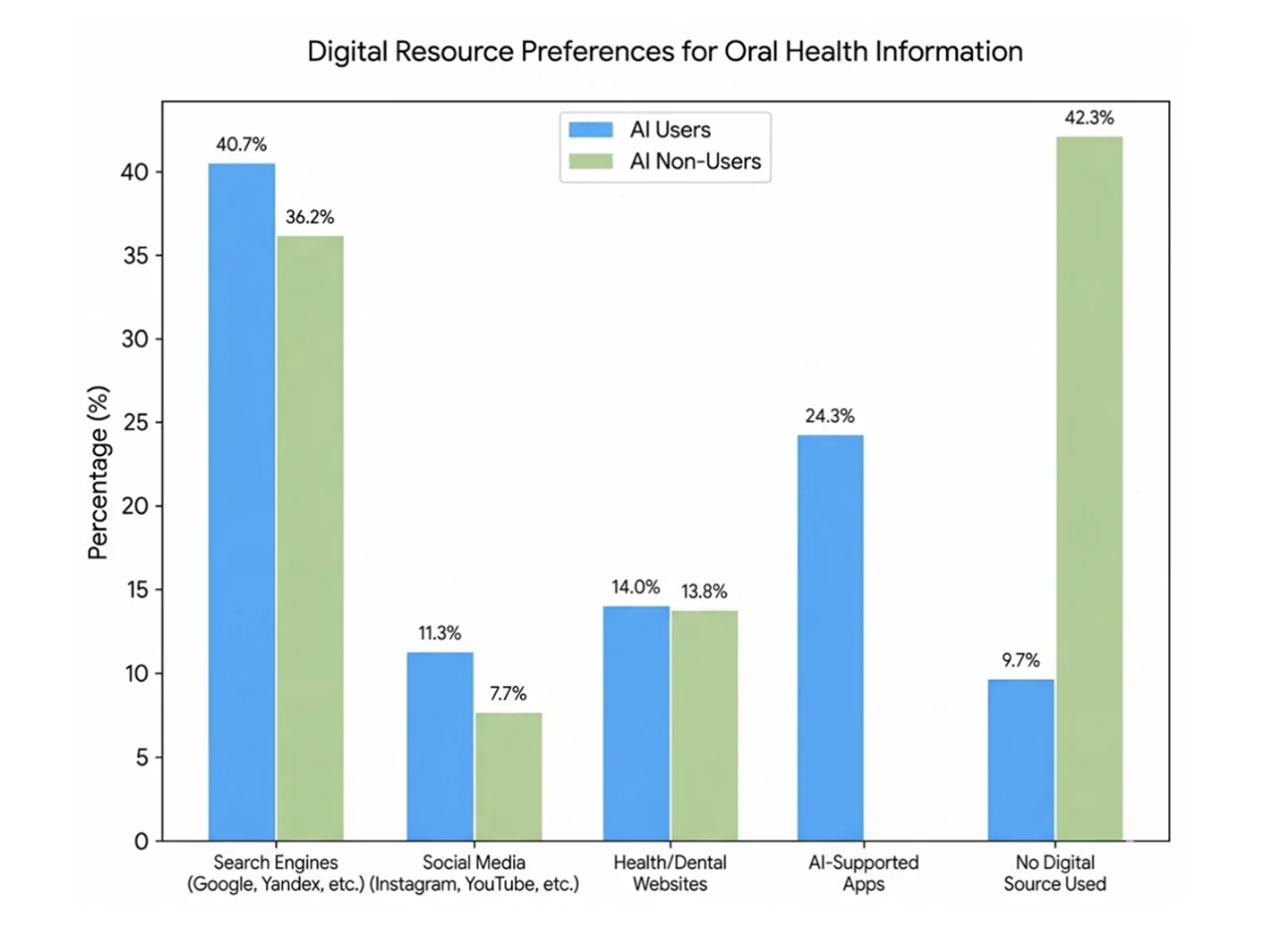
Comparison of digital resource preferences for obtaining oral health information between AI users (n = 300) and non-AI users (n = 130). Bars represent the percentage distribution of participants according to their preferred digital information source.

Trust levels were found to be significantly associated with usage frequency (χ^2^ = 26.6; *p* < 0.01; V = 0.17). Among daily users, the rate of those who found AI-generated information reliable was 44.3%, whereas this rate dropped to 23% among those who used it very rarely. Despite these differences, ‘undecided’ was the most frequent response across all groups, reported by 52.7% of the total sample. This distribution is illustrated in Figure 3. Furthermore, there was no statistically significant relationship between the perceived reliability of AI and the preference for a dentist’s opinion in critical decision-making (χ^2^ = 13.3; *p* ≥ 0.050; V = 0.11).

**Fig 3 Fig3:**
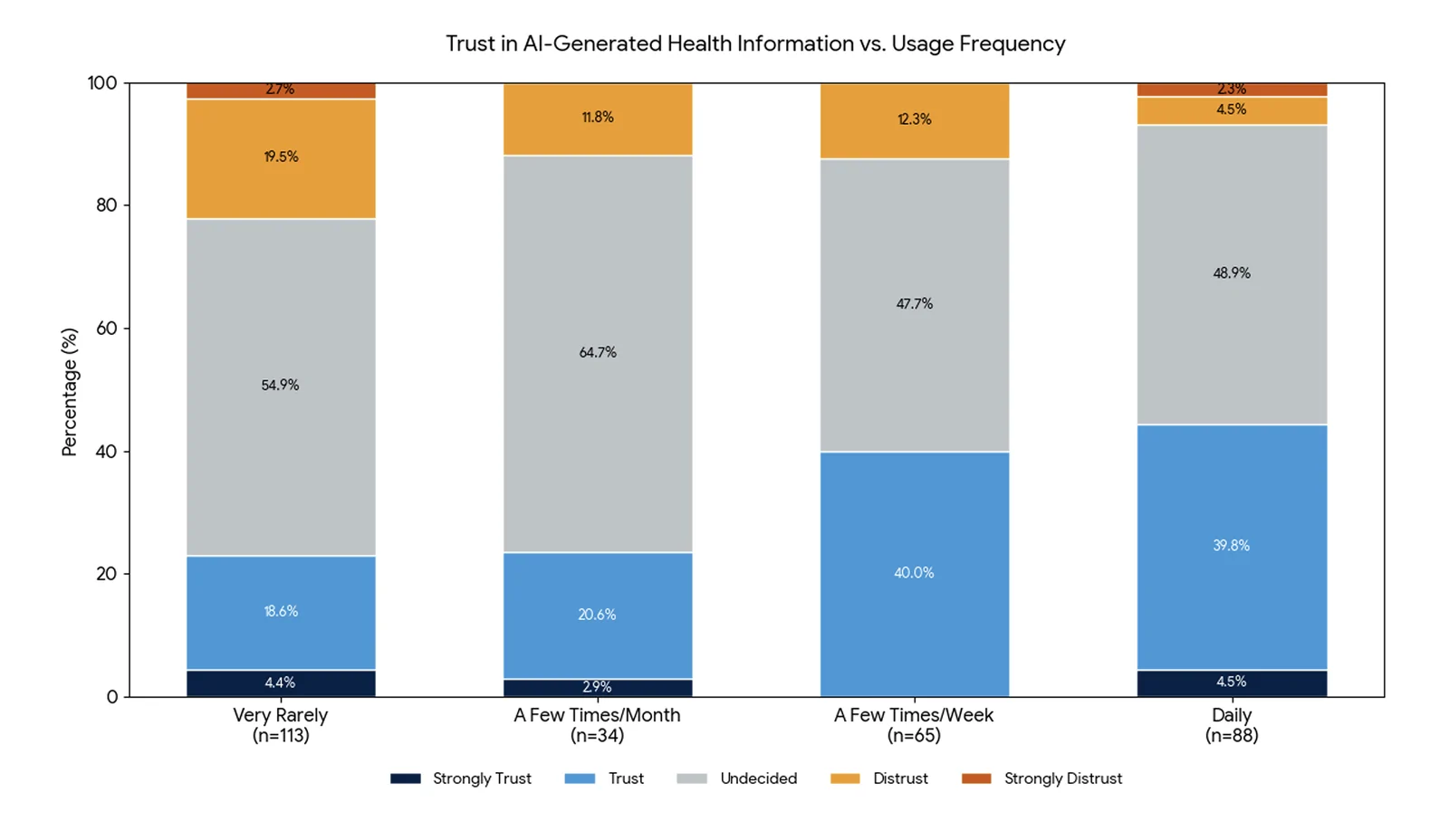
Association between trust in AI-generated information and AI usage frequency among AI users (n = 300). Stacked bars represent the percentage distribution of trust levels across AI usage frequency groups.

A statistically significant relationship was found between education level and the frequency of AI usage (χ^2^ = 72.2; *p* < 0.001; Cramér’s V = 0.24). While the response ‘never use AI’ was predominant among participants with lower education levels (63% among primary school graduates), the rates of daily or several-times-a-week AI usage increased with higher education levels, reaching the highest level among university graduates and participants with postgraduate education (29.3%). Education level was also significantly associated with the perceived reliability of information obtained from AI (χ^2^ = 28.7; *p* = 0.001; Cramér’s V = 0.18). Although the ‘undecided’ response was predominant across all education groups, the proportion of participants considering AI-generated information to be reliable was higher among the high school and university/postgraduate groups (34.8%).

No statistically significant relationship was found between education level and considering AI appropriate for obtaining health-related information (χ^2^ = 16.8; *p* ≥ 0.05; Cramér’s V = 0.14) or preferring the dentist’s opinion over AI in critical oral health decisions (χ^2^ = 12.2; *p* ≥ 0.05; Cramér’s V = 0.12), although the majority of participants across all education levels prioritised the dentist’s opinion. Similarly, no statistically significant relationship was observed between education level and the belief that AI could complement the information provided by dentists (χ^2^ = 10.4; *p* ≥ 0.05; Cramér’s V = 0.11). However, a statistically significant relationship was observed between education level and the willingness to consult an AI-assisted program regarding a treatment plan created by a dentist (χ^2^ = 20.7; *p* = 0.033, Cramér’s V = 0.15). The response ‘I would consider it’ was more common among participants with high school and university/postgraduate education (24% and 30.9%, respectively). Nevertheless, the combined rates of the responses ‘I would not consider it’ and ‘I would definitely not consider it’ were 42.8% among primary school graduates, 46.7% among middle school graduates, 53.2% among high school graduates, and 42.8% among participants with university/postgraduate education.

Prior history of treatment in the periodontology clinic was not significantly associated with preferring the dentist’s opinion over AI in critical oral health decisions (χ^2^ = 3.9; p ≥ 0.05; Cramér’s V = 0.11), indicating a weak association. The proportions of participants responding ‘definitely prefer’ and ‘prefer’ were similar between individuals with and without prior treatment history (34.0% and 23.7% vs 27.1% and 22.7%, respectively). No statistically significant relationship was found between prior treatment history and the intention to consult AI regarding the dentist’s treatment plan (χ^2^ = 6.2; *p* ≥ 0.05; Cramér’s V = 0.14), indicating a weak association. In both groups, the response ‘I would not consider it’ had the highest proportion (45.4% among participants with prior treatment history and 33.0% among those without prior treatment history). Finally, no statistically significant relationship was found between prior treatment history and prioritising the dentist’s opinion over AI when conflicting treatment plans were presented (χ^2^ = 8.5; *p* ≥ 0.05; Cramér’s V = 0.17). In both groups, the responses ‘definitely prioritise’ and ‘prioritise’ regarding the dentist’s opinion were predominant, with rates of 49.5% and 39.2% among participants with prior treatment history and 47.8% and 28.1% among those without prior treatment history, respectively.

## Discussion

AI–supported technologies are increasingly becoming part of everyday health information-seeking behaviour and are creating new forms of interaction between patients and digital information sources.^35^ Although these systems provide rapid and easily accessible medical and dental information, concerns regarding the accuracy, reliability, and clinical appropriateness of AI-generated responses continue to persist.^31^


The growing use of AI in health-related contexts has raised important questions regarding patient trust in AI-generated information and the influence of demographic characteristics such as age, education level, and digital literacy on the adoption of these technologies.^7,19
^ In dentistry, where clinical decision-making is commonly shaped by professional expertise and patient–dentist trust, understanding patients’ attitudes toward AI-assisted applications has become increasingly important.^6^


Our findings demonstrated that AI use was common among the participants, particularly among younger and more highly educated individuals. These findings are consistent with previous studies reporting that demographic characteristics and digital literacy play an important role in the adoption of emerging technologies.^25^


Similarly, Bahadir et al reported that patients with undergraduate and postgraduate education levels exhibited higher trust in AI-based diagnosis systems. The present findings support the view that educational attainment may facilitate both familiarity with and acceptance of AI-assisted technologies in health care settings.^7^ Also, students reported the highest utilisation rates, which may be associated with the widespread use of AI-assisted applications for tasks, assignments, and educational support. Recent studies have similarly shown that AI usage is particularly high among student populations worldwide.^3,30
^


Although AI-assisted tools are widely utilised, uncertainty regarding the reliability and appropriateness of AI-generated information remained the predominant attitude among participants. More than half of the participants reported being undecided about the reliability of information obtained from AI systems. This finding is noteworthy because it suggests that frequent use of AI does not necessarily translate into unconditional trust in the information provided by these systems. Previous studies have similarly emphasised that trust is a complex and multifactorial concept shaped by various psychological, interpersonal, and contextual factors.^28,33
^ Armfield et al described trust as a multidimensional construct whose relationship with health behaviours and professional interactions is not yet fully understood.^4^ In the context of AI systems, trust may differ fundamentally from interpersonal trust because technological trust is primarily based on functionality, predictability, and perceived dependability rather than human intentionality and empathy.^19,22
^ In this respect, the uncertainty observed in the present study may reflect participants’ cautious evaluation of AI-generated health information despite the convenience and accessibility of these technologies.

Another important finding of the present study was that AI use for oral and dental health information appeared to be more limited than its use for general health-related information. Most participants reported either never using AI for oral health information or using it only very rarely. This finding may indicate that, despite increasing familiarity with AI technologies, oral-health-related information-seeking behaviours continue to rely more strongly on traditional and professional information sources. In addition, the highest AI usage was observed among individuals attending the clinic for routine check-ups (18/19; 94.7%). However, this finding should be interpreted with caution because of the small subgroup size. This observation may indicate an association between AI use and preventive health awareness, although further studies with larger samples are needed to confirm this finding.^14^


In the present study, most participants reported that they would consult a dentist when experiencing oral health problems, and a substantial majority prioritised the dentist’s opinion when AI-generated recommendations conflicted with professional treatment plans. Furthermore, trust in AI-generated information was not significantly associated with preferring AI over dentists in important oral health decisions. These findings are consistent with previous literature emphasising that health professionals continue to represent the primary source of reliable information for patients.^2,15
^ Similarly, Bahadir et al reported that patients generally accepted AI-assisted applications in dentistry, while professional trust in dentists remained central to clinical decision-making.^7^ In the present study, even participants who reported relatively higher levels of trust in AI generally continued to prioritise professional clinical expertise in treatment-related decisions. This pattern suggests that AI is currently perceived primarily as a complementary informational resource rather than an independent clinical authority.^18^


An additional noteworthy finding was the association between AI usage frequency and trust perception. Participants who used AI applications more frequently tended to report higher levels of perceived reliability, suggesting that greater familiarity with these technologies may contribute to increased trust. Nevertheless, the persistence of high indecision rates even among frequent users suggests that familiarity alone may not be sufficient to establish full confidence in AI-assisted health information.

The present study has several important strengths. First, the sample size for the AI-user subgroup was determined through an *a priori* power analysis, ensuring adequate statistical power for the planned analyses. Second, the questionnaire underwent a formal content-validity assessment using Lawshe’s methodology, with evaluation by an expert panel and calculation of CVR and CVI values, supporting the adequacy of the instrument’s content validity. Finally, in addition to statistical significance testing, effect sizes (Cramér’s V) were reported to facilitate interpretation of the practical relevance of the observed associations.

Several limitations of the present study should be acknowledged. First, the cross-sectional design limits causal interpretation of the observed associations. Second, the study population consisted of patients from a single-centre university clinic, which may limit the generalizability of the findings to broader populations. Third, participants’ digital literacy was not assessed. As digital literacy may influence both the use and interpretation of AI-assisted applications, it may also have affected participants’ responses. In addition, the findings were based on self-reported responses and may therefore be affected by response bias. Finally, rapidly evolving AI technologies and changing public familiarity with these systems may influence attitudes and trust perceptions over time. Future studies should include multicenter or community-based populations to improve the generalisability of the findings, evaluate changes in patients’ perceptions as familiarity with AI-assisted applications evolves over time, and further validate the questionnaire in different populations.

In conclusion, AI-assisted applications appear to have become an important component of contemporary health information-seeking behaviour, particularly among younger and more educated individuals. However, patients remain cautious regarding the reliability of AI-generated information and continue to prioritise professional dental expertise in critical clinical decisions. These findings support the view that AI currently functions primarily as a supportive informational resource rather than a replacement for professional dental care.

### Acknowledgements

#### Data availability statement

The data generated and analysed during this study are available from the corresponding author upon reasonable request.

#### Funding statement

This research did not receive any specific grant from funding agencies in the public, commercial, or not-for-profit sectors.

#### Conflict of interest

The authors declare that they have no conflicts of interest related to this study.

#### Ethics approval statement

The study was approved by the University of Health Sciences Gulhane Training and Research Hospital Clinical Research Ethics Committee (approval no: 2026/109).

#### Permission to reproduce material from other sources

No previously published figures, tables, or materials were reproduced in this manuscript; therefore, no permissions were required.

#### Clinical trial registration

This study did not involve a clinical trial, and therefore registration was not required.

#### AI statement

ChatGPT-5.5 and Gemini 1.5 were used for language editing and preparation of graphical materials during manuscript preparation. All figure values were verified by the authors against the original study data. All content, analyses, interpretations, and graphical materials were critically reviewed and approved by the authors.

## References
